# Trajectories of perceived household chaos and youth health outcomes across the COVID-19: insights from individual, dyadic, and triadic family analyses

**DOI:** 10.3389/frcha.2026.1717371

**Published:** 2026-06-22

**Authors:** Chunyuan Xi, Jacinda Kay Dariotis, Dana Ann Eldreth, Iffat Noor, Rebecca Lee Smith

**Affiliations:** 1 Department of Human Development and Family Studies, The Family Resiliency Center, College of Agricultural, Consumer & Environmental Sciences, The University of Illinois at Urbana-Champaign, Urbana, IL, United States; 2 Department of Biomedical and Translational Sciences, Carle Illinois College of Medicine, The University of Illinois at Urbana-Champaign, Urbana, IL, United States; 3 Carl R. Woese Institute for Genomic Biology, The University of Illinois at Urbana-Champaign, Urbana, IL, United States; 4 Department of Health and Kinesiology, College of Applied Health Sciences, The University of Illinois at Urbana-Champaign, Champaign, IL, United States; 5 Department of Pathobiology, College of Veterinary Medicine, The University of Illinois at Urbana-Champaign, Urbana, IL, United States

**Keywords:** adolescents, COVID-19, families, household chaos, mental health, pandemic preparedness, resilience, sleep disturbance

## Abstract

**Introduction:**

The COVID-19 pandemic profoundly disrupted youth development; however, limited studies have explored the diverse patterns of household environments and their implications for youth and family well-being. This study aimed to identify trajectories of household chaos during the COVID pandemic and to investigate their associations with youth mental and physical health outcomes.

**Methods:**

Data were collected from 506 adults (*M*_age_ = 42.8, *SD*_age_ = 9.15) and 93 youth (*M*_age_ = 14.5, *SD*_age_ = 1.63). Using a retrospective cross-sectional design, participants reported their experiences across the COVID-19 pandemic. Parents and youth provided reports of household chaos for pre-, mid-, and post-COVID timepoints and on youth health-related outcomes at pre- and post. Latent Class Growth Analysis (LCGA), Analysis of Covariance, and Analysis of Variance were used.

**Results:**

Using parent and youth self-report data, LCGA identified three trajectories of household chaos (low-chaos “thriving,” 35.2%; high-chaos “worsening,” 24.5%; and moderate-chaos “resilience,” 40.3%). Dyadic and triadic analyses revealed differing parent and youth household chaos perceptions. Within the same family, younger youth showed greater concordance with parents compared to older youth. Youth on the high-chaos worsening trajectory exhibited higher levels of behavioral problems, emotional-behavioral dyscontrol, emotional distress-anxiety, sleep disturbance, and lower levels of positive affect and well-being, compared to one or both other trajectory groups.

**Discussion:**

Findings highlight meaningful differences in parent and youth pandemic-related household chaos experiences, which are linked to youth health outcomes. Public health intervention and prevention programs should account for both shared and nonshared family experiences to better support youth well-being as part of future pandemic preparedness.

## Introduction

### COVID-19 impact on youth mental and physical well-being

There has been an increased global concern for worsening youth well-being, including mental and physical health, throughout and after the COVID-19 pandemic (1, 2). Youth, who are highly sensitive to social dynamics and heavily rely on peer connections for emotional support (3), faced unique challenges due to widespread pandemic-related disruption in schooling, leisure activities, peer networks, and family routines (4). School closures, social distancing, and the cancellation of extracurricular activities contributed to increased feelings of loneliness and prolonged periods of physical and social challenges (5). A substantial body of meta-analytic and review studies has documented the heightened psychological impact of the pandemic on children and youth globally, including the increased prevalence of emotional disorders. Rates include one in 4 or 5 affected youth, such as anxiety (20.5%–23.2%) and depression (22.8%–25.2%), depending on the condition (6, 7). Beyond its effects on mental health, research has also linked the COVID-19 pandemic and lockdown to worsened youth sleep quality, particularly in terms of disruptions to their daily routines, wake-up times, with an estimated 38.9% of youth affected by insomnia (8).

### Family disruptions as a risk factor for youth maladjustment

In addition to its direct psychological effects, the COVID-19 pandemic substantially altered youths’ living environments, particularly through household chaos (HHChaos), a construct referring to disorganization or environmental confusion within the family (9). During the COVID-19 period, parents worldwide experienced significant stressors, including responding to their children's transition to remote learning, loss of employment, and illness or death of loved ones (10). These losses contributed to disruptions in family support, routines, and financial stability, ultimately exacerbating HHChaos (11). According to family systems theory, disruptions within the family system are expected to influence children's adjustment (12). Specifically, increased HHChaos, characterized by irregular family routines, excessive noise levels, and overcrowding, may be stressful for families and linked to adverse emotional and behavioral outcomes in children (13–15). Borrowing from behavioral genetics, members of the same family may have similar and different experiences of stressors and disruptions, speaking to the need to explore shared and non-shared experiences (16). As will be discussed in more depth later, how families manage disruptions may be malleable (17) and could provide an avenue for intervening and buffering youth against disruptions and related negative health outcomes.

### HHChaos and youth mental and physical health

A growing body of empirical evidence has examined the associations between HHChaos and mental health outcomes for children in middle childhood and early adolescence (18, 19). For example, HHChaos at age 5 has been associated with greater children's internalizing and externalizing problems by age 7 (20). During the COVID-19 pandemic, HHChaos has been positively connected with emotional distress (e.g., anxiety/withdrawal, fear) in preschool-aged children (21). Studies among early youth have identified positive associations between HHChaos and depression, internalizing symptoms, and disruptive behaviors (18, 22, 23). Research examining early, mid, and late adolescence in a single study—particularly teenagers who are experiencing increasing autonomy and emotional regulation—remains limited, and findings from existing studies are inconsistent. For instance, some studies have reported significant positive associations between HHChaos and youth internalizing and externalizing problems (24, 25), whereas other scholars did not find a significant correlation between parent-reported chaos and youth's anxiety or depressive symptoms (26). Also, another study did not demonstrate the main effect of HHChaos on youth internalizing or externalizing problems, but rather that the relationship between HHChaos and behavioral problems depends on the child's emotional reactivity [i.e., how children express emotions in response to events; (27)]. In the same study, no significant correlation was found between HHChaos and youth internalizing problems, a significant association was found with externalizing problems. These mixed and discrepant findings underscore the need for further investigation to clarify these associations.

Beyond the association between HHChaos and children's mental health, research has also linked HHChaos to physical health, particularly sleep quality. Youth frequently experience insufficient sleep and excessive daytime sleepiness, with insomnia affecting 7%–40% of youth depending on different diagnostic criteria (28). Prevalent factors impacting sleep disturbance among youth include academic demands, extracurricular activities, and excessive technology use (29, 30). Prior research suggests that a lack of family routines is associated with poorer sleep quality in early school-aged children (31). Studies investigating HHChaos and youth sleep disturbance are limited, with some evidence for a positive relationship between family disorganization and youth sleep problems (26). Given the well-documented links between sleep disturbance and youth affective functioning, depressive symptoms, and externalizing problems (32, 33), it is crucial to examine both youth mental health and sleep disturbance in relation to HHChaos and to consider the potential for HHChaos as a target for intervention.

### Need for person-centered approaches and youth perspectives

Pre-COVID pandemic research on HHChaos and youth behavior has primarily employed a variable-centered approach, overlooking the potential for distinct patterns of family chaos. One study identified factors that characterize the home environment, including HHChaos, parent stress, and quality of parent-child interactions (34). Findings from this study point to the importance of exploring chaos as a dynamic construct, particularly during unforeseen societal disruptions such as the COVID-19 pandemic. Additionally, most prior studies have relied on caregiver-reported HHChaos to predict youth outcomes, often neglecting youth perspectives. In a notable exception, one recent study found no significant association between youth and caregiver reports of HHChaos within the dyad (25). This finding highlights the importance of incorporating youth perspectives on their living environment in studies. Understanding these differing perspectives is critical for obtaining a comprehensive picture of the household dynamics. Identifying HHChaos change patterns and their associations with youth well-being can further inform post-pandemic recovery efforts and strengthen family resilience against future challenges.

### Importance for future pandemic preparedness

Moving forward, studies conducted about COVID-19 provide important insights into how youth rely on family contexts for future-oriented guidance during periods of heightened social uncertainty and disruption. Taking a life-course (35) and person-in-environment perspectives (36, 37), the COVID-19 pandemic offers an opportunity to reconsider what constitutes “normal” day-to-day experiences for youths’ present and future selves.

Specifically, a better understanding of how parents and youth perceive changes in the home environment—and how parents and youth, as well as older and younger siblings within the family system, experience shared and non-shared environments—may help clarify how these processes are linked to youth developmental outcomes. Such insights could provide a unique rationale for identifying which families may benefit most from support and, within families, how to tailor support for individual youth based on their distinct experiences and the ways these experiences relate to later outcomes. These considerations may be especially relevant for youth navigating future structural or contextual challenges.

### The present study

The current study sought to narrow gaps in the literature by adopting a person-centered approach to HHChaos and investigating its association with youth outcomes. This study was guided by three research goals. First, the study identified unique latent class growth trajectories of HHChaos across pre-, mid-, and post-COVID-19pandemic timepoints, using both adult and youth-reported data. Given the limited existing literature on varying HHChaos trajectory profiles during the COVID pandemic period, it was hypothesized that multiple HHChaos trajectories would emerge, some improving and others worsening, reflecting the diverse ways in which families and individuals adapted to the COVID-19 pandemic (38). Second, the study examined parents’ and youth perceptions of HHChaos using dyadic and triadic data, assessing whether family members report consistent or different patterns of change across the COVID-19 pandemic. Based on prior research, it was hypothesized that parents and their children in the same household would experience HHChaos differently. Third, the study investigated the links between distinct HHChaos trajectories and youth mental health and sleep outcomes, including behavioral problems, emotional distress and anxiety, well-being, and sleep disturbance. As the existing evidence supports the relationship between HHChaos and youth behavioral functioning, it was expected that high chaos trajectories would be significantly associated with poorer youth mental and physical health outcomes.

## Methods

### Participants

The current study is part of a larger mixed-method study focusing on COVID-19 pandemic responses and adaptations by parents, youth, and childcare providers [see study protocol for details; (10)]. A total of 506 adults (*M*_age_ = 42.8, *SD*_age_ = 9.2), of whom 19% did not have a child 17 years or younger, and 43.1% did not have a second child 17 years or younger. A total of 93 youth (*M*_age_ = 14.5, *SD*_age_ = 1.6) representing 82 families participated in the study. Within the families with multiple children, we designated the oldest child under 17 years as the “older youth” and the second oldest child under 17 years as the “younger youth.” Subsamples included 56 parent–older youth dyads and 26 parent–younger youth dyads (Parents: *M*_age_ = 44.5, *SD*_age_ = 6.3; Older youth: *M*_age_ = 14.6, *SD*_age_ = 1.6; Younger youth: *M*_age_ = 14.1, *SD*_age_ = 1.6), and 20 parent–youth–youth triads (Parents: *M*_age_ = 46.3, *SD*_age_ = 6.1; Youth: *M*_age_ = 13.9, *SD*_age_ = 1.7).

### Procedure

Participants were recruited primarily from a large Midwestern state through established databases of participants from other studies, local schools, community venues, and in-person events. Statewide COVID-19 mitigation measures were implemented beginning in March 2020, including the closure of public and private K–12 schools, the suspension of on-site dining, the closure of non-essential businesses, and a mandatory stay-at-home order effective March 21, 2020 (39, 40). These restrictions were gradually lifted, with all industries fully reopened by June 11, 2021 (41). Participants were asked about current experiences (2022–2023, post-COVID) and to recall their experiences before COVID-19 was declared a worldwide pandemic by the World Health Organization (42) and a national emergency in the United States (43) in March 2020; (2-years ago, before COVID-19, March 2020; pre-COVID) and one year into the pandemic (March 2021; mid-COVID) when vaccines became available to everyone aged 16 an older within the United States (44) for selected scales and subscales. The surveys took ∼40 min for adults and ∼20 min for youth to complete. This study was approved by the [Anonymized]. The participants provided consent to be part of the parent study.

### Measures

#### Household chaos

HHChaos was evaluated using the Confusion, Hubbub, and Order Scale [CHAOS; (45)], a 15-item measure rated on a 4-point Likert scale from 1 (*very much*) to 4 (*not at all*). Example items include: “*First thing in the day, we have a regular routine at home”* and “*Our home is a good place to relax*.” Total scores were calculated by summing all items, with higher scores indicating greater levels of disorganization, confusion, and noise within the home environment. Both adults and youth provided ratings at pre-, mid-, and post-COVID time points. Cronbach's alpha ranged from 0.73 to 0.79 across reporters and time points.

#### Problem behaviors

Youth behavioral problems were assessed using the Strengths and Difficulties Questionnaire [SDQ; (46)]. This 25-item measure includes four problem behavior subscales: Hyperactivity, Emotional Symptoms, Conduct Problems, and Peer Problems. Each subscale is rated using a 3-point Likert scale (0 = *not true*, 1 = *somewhat true*, 2 = *certainly true*). These four subscale scores were summed to create a Total Difficulties score, with higher scores indicating greater behavioral problems. Adults provided ratings about pre-, mid-, and post-COVID time points, while youth self-reported about pre- and post-COVID. The Total Difficulties scale Cronbach's alpha ranged from 0.75 to 0.83 across reporters and time points.

#### Youth emotional and physical wellbeing

Several instruments from the Patient-Reported Outcomes Measurement Information System (PROMIS) were used to assess youth emotional and physical wellbeing. Youth self-reported on the following constructs at the post-COVID time point.

Emotional-Behavioral Dyscontrol (47) is an 8-item measure evaluating emotional regulation and behavioral control. Responses were recorded using a 5-point Likert scale from 1 (*never*) to 5 (*always*). The EBD total score was calculated by summing all items with higher scores indicating greater emotional and behavioral dyscontrol. Cronbach's alpha value was 0.91.

Emotional Distress-Anxiety (48) is an 8-item scale measuring anxiety and emotional distress in youth. Items were rated on a 5-point Likert scale ranging from 1 (*never*) to 5 (*almost always*). The total score was calculated by summing scores for all items with higher scores indicating a greater level of pediatric emotional distress and anxiety. Cronbach's alpha value was 0.90.

Sleep-Related Disturbance (49) is a 6-item measure evaluating sleep quality and disturbances. Each item was rated on a 5-point Likert scale, ranging from 1 (*not at all*) to 5 (*very much*). The total score was calculated by summing five items. Higher scores indicated greater severity of sleep disturbances. Cronbach's alpha value was 0.73.

Positive Affect and Well-Being (47) is a 9-item measure evaluating positive affect and overall well-being. Youth responded to a 5-point Likert ranging from 1 (*never*) to 5 (*almost always*). The total score was calculated by summing all items, with higher scores indicating a greater sense of positive affect and well-being. Cronbach's alpha value was 0.90.

### Data analyses

Data analyses were conducted in SPSS 29.0 and Mplus 8.3 using the maximum likelihood estimator. Descriptive statistics were conducted for the main variables.

**Figure 1:**
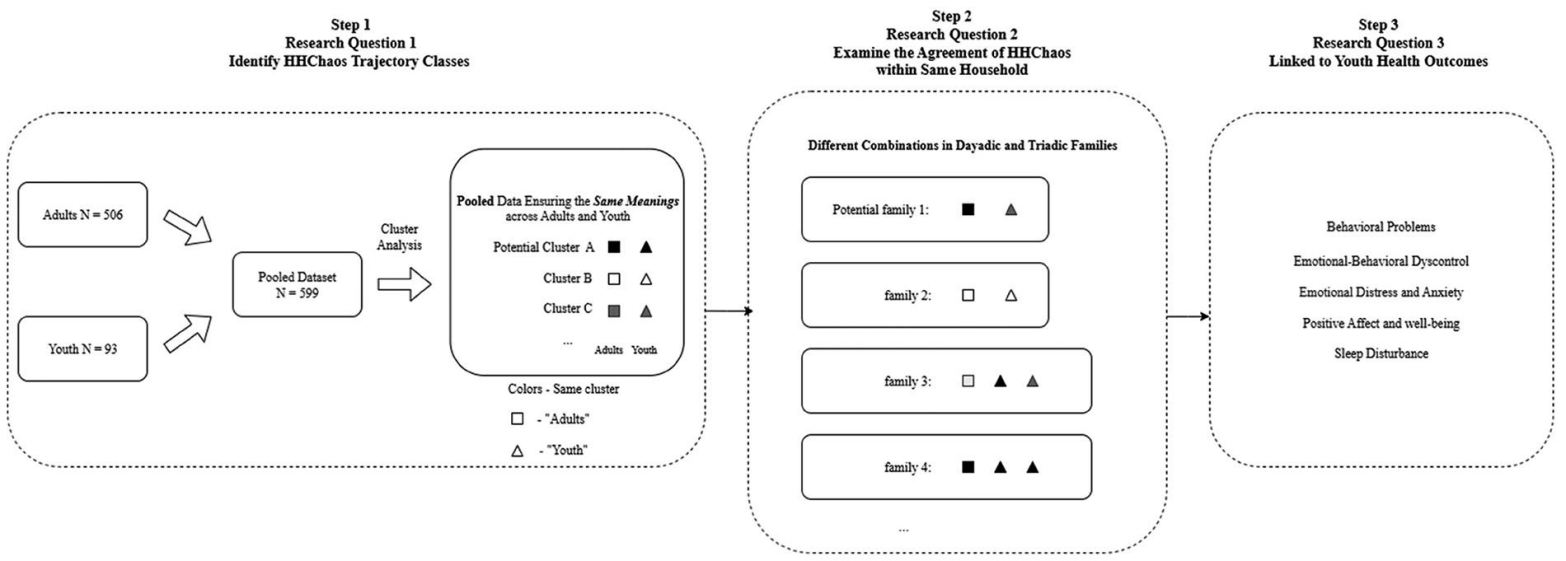
Flowchart of research question, justifying the pooled data to ensure the consistent cluster meaning across respondents and time points.

Latent Class Growth Analysis (LCGA) was used to identify HHChaos trajectory clusters. LCGA does not assume that individual variations align from a single population trajectory (50), thereby enabling the detection of multiple unique patterns of HHChaos within a population over time. Trajectory analyses were run pooling the full sample of adults and youth (*N* = 599) to ensure a consistent definition of class trajectories across groups (see the flowchart in Figure 1). The first slope loading was fixed at 0, and the subsequent slope coefficients were fixed at 1 and 2 for T2 and T3, respectively. Then, within-class variances were fixed to zero, which allowed a clearer identification of classes and reduced computational burden (51). The model fit indices used for model selection included the smallest Akaike (AIC) and Bayesian information criteria value (BIC); a significant Lo-Mendell-Rubin likelihood ratio test (LMR-LRT) and bootstrap likelihood ratio test (BLRT); and entropy values [range: 0 to 1; >0.80 means accurate classification; (52)].

Cross-reporting agreement analyses were then conducted to assess whether parents and youth within the same family were classified into the same HHChaos trajectory using dyadic and triadic samples. Analysis of covariance (ANCOVA) and analysis of variance (ANOVA) were performed to assess whether significant differences existed in youth outcomes across the identified HHChaos trajectory classes. For ANCOVA, the pre-COVID pandemic youth behavioral problem was added as the covariate.

## Results

### Descriptive analysis

Table 1 displays participant demographic characteristics for the full sample and dyadic and triadic subsamples. Table 2 presents descriptive statistics for HHChaos and youth behavioral outcomes. Results of ANCOVA, ANOVA, and paired *t*-test revealed significant changes in HHChaos and youth outcomes across time.

**Table 1:** Adult, youth, dyadic, and triadic sample demographic characteristics.

Measures	Adult sample (*N* = 506)		Youth sample *(N* = 93)		Dyadic sample						Triadic sample			
					Parent (*N* = 62)		Older youth (*N* = 56)		Younger youth (*N* = 26)		Parent (*N* = 20)		Youth (*N* = 40: 20 of older youth and 20 of younger youth)	
	%	*N*	%	*N*	%	*N*	%	*N*	%	*N*	%	*N*	%	*N*
Gender														
Female	92.3	467	55.9	52	82.3	51	66.1	37	42.3	11	70	14	55	22
Male	7.5	38	43	40	17.7	11	32.1	18	57.7	15	30	6	45	18
Genderqueer/Gender non-confronting	0.2	1	1.1	1	0	0	0	0	0	0	0	0	0	0
Missing	0	0	0	0	0	0	1.8	1	0	0	0	0	0	0
Race														
American Indian or Alaska Native	1	5	0	0	0	0	0	0	0	0	0	0	0	0
Asian	5.7	29	4.3	4	8.1	5	5.4	3	3.8	1	5	1	5	2
Black or African American	10.7	54	6.5	6	4.8	3	5.4	3	3.8	1	5	1	5	2
White	81.4	412	76.3	71	87.1	54	78.6	44	80.8	21	90	18	80	32
Something else	1.2	6	8.6	8	0	0	8.9	5	11.5	3	0	0	10	4
Missing	0	0	4.3	4	0	0	1.8	1	0	0	0	0	0	0
Education level														
High school degree or general equivalency diploma	4.5	23	–	–	3.2	2	–	–	–	–	0	0	–	–
Some college but no degree	11.9	60	–	–	3.2	2	–	–	–	–	5	1	–	–
Associate's degree	8.3	42	–	–	9.7	6	–	–	–	–	5	1	–	–
Bachelor's degree	29.6	150	–	–	95.5	22	–	–	–	–	50	10	–	–
Graduate degree	45.7	231	–	–	48.4	30	–	–	–	–	40	8	–	–
Marital Status														
Single or widowed	15.2	77	–	–	4.8	3	–	–	–	–	0	0	–	–
Married or civil union	78.1	395	–	–	91.9	57	–	–	–	–	95	19	–	–
Separated or divorced	6.7	34	–	–	3.2	2	–	–	–	–	5	1	–	–
Annual Household Income														
$50,000 or more	74.7	378	–	–	87.1	54	–	–	–	–	80	16	–	–
$27.000–$49,000	11.9	60	–	–	8.1	5	–	–	–	–	20	4	–	–
$26,000 or less	4.7	24	–	–	3.2	2	–	–	–	–	0	0	–	–
Missing	8.7	44	–	–	1.6	1	–	–	–	–	–	–	–	–

**Table 2:** Descriptives of main variable*s.*

Variables	Pre-COVID			Mid-COVID			Post-COVID			ANOVA, *t*-test Results
	*N*	Mean	SD	*N*	Mean	SD	*N*	Mean	SD	
HHChaos	571	29.5	6.6	571	30.6	6.9	571	29.3	6.6	*F* = 5.77, *p* = 0.003
SDQ^a^_Youth 1_Adult reports	234	8.8	5.6	–	–	–	234	9.5	6.4	*t* = −3.61, *p* < 0.001
SDQ^a^_Youth 2_Adult reports	133	9.3	5.8	–	–	–	133	9.5	5.9	*t* = −0.57, *p* > 0.05
SDQ^a^_Youth_Self reports	82	13.1	5.6	–	–	–	82	14.2	6.5	*t* = −2.08, *p* < 0.05
Emotional-Behavioral Dyscontrol	–	–	–	–	–	–	76	21.1	6.5	–
Emotional Distress-Anxiety	–	–	–	–	–	–	78	20.6	6.7	–
Sleep Disturbance	–	–	–	–	–	–	76	11.0	3.9	–
Positive Affect and Well-Being	–	–	–	–	–	–	76	32.6	6.5	–

a SDQ, Strengths and Difficulties Questionnaire.

### Identifying trajectories of HHChaos for adults and youth

**Figure 2:**
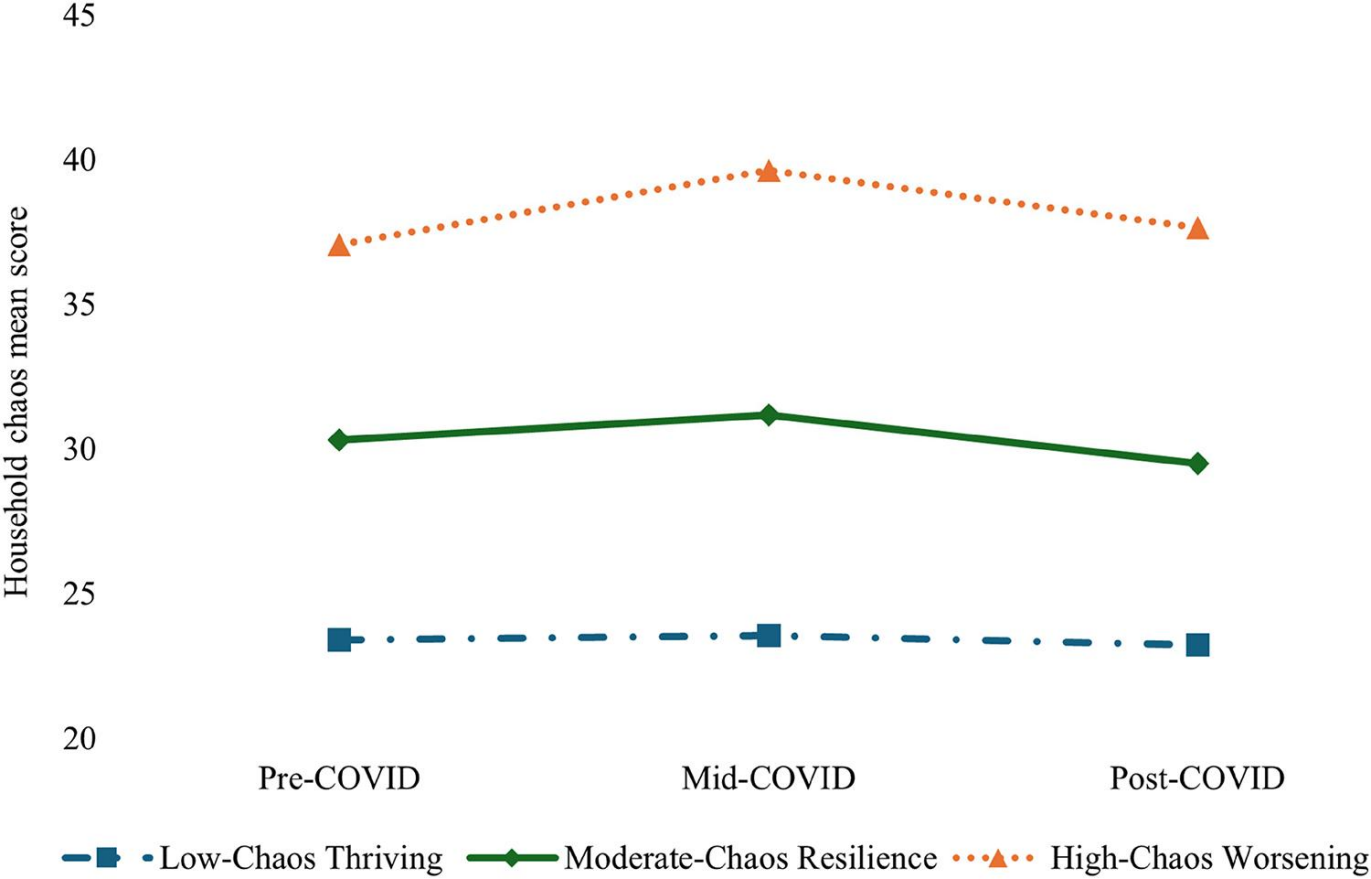
Mean score changes of HHChaos across the COVID.

To answer the first research question–HHChaos patterns across the COVID-19 pandemic–LCGA was conducted to capture distinct HHChaos trajectories. LCGA was performed on data across adults and youth. Model fit indices confirmed that a three-class model was the best model [AIC = 10,492.85, BIC = 10,540.67, Entropy = 0.80, LMR-LRT(*p*) = 0.0001, LRT (*p*) < 0.0001]. Participants in the smallest trajectory class (*N* = 140; 24.5%) had the highest HHChaos across all three time points, with a high value at pre (*M* = 37.1) that increased at mid (*M* = 39.6) and post (*M* = 37.7). Given that their post-COVID pandemic chaos was higher than that at pre-COVID and they experienced elevated chaos compared to other classes pandemic, we classified this trend as “*High-Chaos Worsening*.” The next largest trajectory (*N* = 201; 35.2% of the sample) had the lowest HHChaos throughout the pandemic (*M* = 23.4) and experienced lower post-COVID pandemic HHChaos levels (*M* = 23.2) relative to pre-COVID pandemic levels, we label this class as “*Low-Chaos Thriving*” (better outcomes after adversity). The largest trajectory (*N* = 230; 40.3%) started with moderate HHChaos at pre (*M* = 30.3), experienced an increase at mid (*M* = 31.2), and recovered to pre-levels at post-COVID (*M* = 29.5). We label this trajectory “*Moderate-Chaos Resilience*.” Figure 2 graphically depicted the mean score changes on HHChaos across groups. The distribution of class membership was fairly similar for adults (with 23.82% in “High-Chaos Worsening”, 35.93% in “Low-Chaos Thriving”, and 40.25% in “Moderate-Chaos Resilience”) and youth (28.57%, 30.95%, and 40.48%, respectively).

### HHChaos trajectory agreements between parents and youth using dyadic and triadic samples

To address the second research question, we examined the extent to which parents and youth within the same family similarly or differentially experienced HHChaos throughout the COVID pandemic. After identifying trajectory classes, class membership was assigned to each individual. Cross-reporting agreement analyses were then conducted to assess whether parents and youth within the same family were classified into the same HHChaos trajectory. Shared trajectory membership was used as an indicator of similarity in perceived household chaos.

As shown in Table 3, in the dyadic samples (*N* = 56, parent–older youth pairs), 33% of older youth shared the same trajectory as their parents when parents were classified as low-chaos thriving, whereas 67% of their children were members of high-chaos worsening or moderate-chaos resilience, suggesting they perceived a higher level of HHChaos than their parents. When parents were classified as high-chaos worsening, 43% of their older children experienced the same trajectory, 57% were in the moderate-chaos resilience trajectory, and none were in the low-chaos thriving trajectory. In addition, when parents were in the moderate-chaos resilience trajectory, 60% of their older children were members of the same trajectory, followed by 27% in low-chaos thriving and 13% in high-chaos worsening trajectories.

**Table 3:** Shared household chaos trajectory classes between adults and youth.

Dyadic pairs (Adult–older youth, *N* = 56)				
Adult		Older youth		
		Low-chaos thriving	High-chaos worsening	Moderate-chaos resilience
	Low-Chaos Thriving	**33** **.** **33%**	25.93%	40.74%
	High-Chaos Worsening	0.00%	**42** **.** **90%**	57.10%
	Moderate-Chaos Resilience	26.70%	13.30%	**60** **.** **00%**
Dyadic pairs (Adult–younger youth, *N* = 26)				
Adult		Younger Youth		
		Low-Chaos Thriving	High-Chaos Worsening	Moderate-Chaos Resilience
	Low-Chaos Thriving	**80** **.** **00%**	10.00%	10.00%
	High-Chaos Worsening	11.10%	**66** **.** **70%**	22.20%
	Moderate-Chaos Resilience	28.60%	28.60%	**42** **.** **90%**
Triadic pairs (Adult–older Youth–younger youth, *N* = 20)				
Only 20% of youth shared the same trajectory classes with their parents.				

*Note*. Bolded percentages indicate cases in which the parent and child were classified into the same trajectory class.

Agreements were higher for parents and their younger children (*N* = 26 pairs) compared to older children in two HHChaos trajectory classes. Specifically, agreement percentages were 80% and 33% in low-chaos thriving and 67% and 43% in high-chaos worsening trajectories for younger children and older children, respectively. This indicates that younger children were more likely to share their parents’ perceptions, particularly for HHChaos low-chaos thriving membership. For the moderate-chaos resilience class, 43% of younger children agreed with parents (vs. 60% of older children).

In the triadic sample (*N* = 20; adult–older youth–younger youth), only 20% of parents and their two children shared the same trajectories. Furthermore, discrepancies between older children and younger siblings were observed. Within a triad, younger children were more likely to align with their parents’ perceptions of HHChaos than their older siblings.

### Association between household trajectories and youth outcomes

**Figure 3:**
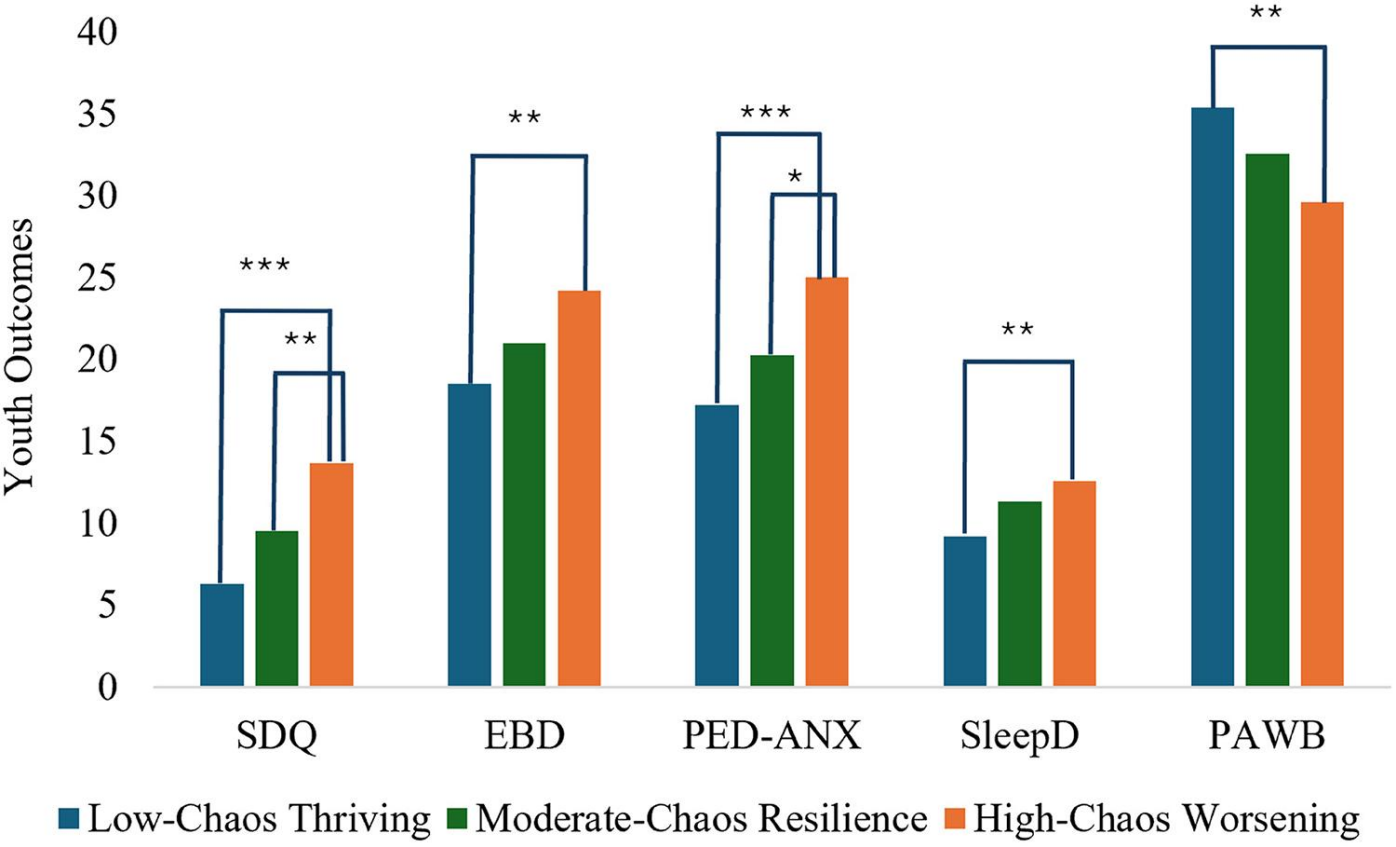
ANCOVA and ANOVA results on the association between household trajectories and youth outcomes. SDQ, Strengths and Difficulties Questionnaire; EBD, Emotional-Behavioral Dyscontrol; PED-ANX, Emotional Distress-Anxiety; SleepD, Sleep Disturbance; PAWB, Positive Affect and Well-Being. *p < .05; **p < .01; ***p < .001.

As for the third research question–how trajectories relate to youth outcomes–was answered using ANCOVA and ANOVA. As shown in Figure 3, analyses revealed significant differences in youth outcomes across chaos trajectory classes. For older children in the household, adult-reported behavioral problems were significantly higher in the high-chaos worsening class compared to other trajectories (*F* = 9.73, *p* < 0.001), after controlling for pre-COVID reports about youth behavioral problems. In contrast, younger children showed no significant differences, and youth-reported behavioral problems did not vary significantly across chaos trajectories. Additional significant group differences were observed for emotional-behavioral dyscontrol (*F* = 4.88, *p* = 0.01) and sleep disturbance (*F* = 4.94, *p* = 0.01), with youth in the high-chaos worsening trajectory exhibiting higher levels of these outcomes compared to low-chaos thriving youth. However, no significant differences were found between the high-chaos worsening and moderate-chaos resilience trajectories. Furthermore, high-chaos worsening youth showed higher emotional distress-anxiety than those in the low-chaos thriving and moderate-chaos resilience trajectories (*F* = 9.76, *p* < 0.001). By contrast, positive affect and well-being were significantly higher for low-chaos thriving youth compared to high-chaos worsening youth (*F* = 5.09, *p* = 0.01). These findings suggest that youth in households on the high-chaos worsening class trajectory experience an increased risk of behavioral problems, emotional distress, anxiety, and sleep disturbance.

## Discussion

HHChaos has been consistently associated with youth developmental outcomes, particularly during the sensitive period of the COVID-19 pandemic, when both parents and children were faced with widespread and unexpected challenges. However, to our knowledge, little is known about how household functioning trajectories shifted during this period, especially from the perspectives of youth, who may perceive and interpret their environment differently than adults, even within the same household and across siblings of different ages. This study identified three distinct household functioning trajectories in response to the historical stressor of the pandemic, highlighting important perceptual and experiential differences among parents and youth. These findings underscore the role of HHChaos in affecting youth mental health and sleep outcomes, emphasizing the critical importance of capturing family dynamics from multiple perspectives and informing the need for developmentally sensitive, tailored, family-based interventions.

### Trajectories of HHChaos across the COVID-19 pandemic

Three distinct trajectories of HHChaos experiences across the COVID-19 pandemic emerged for adults and youth, with 40% experiencing moderate levels with resilience despite HHChaos experiencing hardship. This resilience may be attributable to the reopening of schools, increased access to community resources, and reduced uncertainty related to work and personal life. In the low-chaos thriving class (35%), adults and youth reported significantly lower family disorganization and confusion, with an overall downward trend in chaos following the COVID pandemic. In contrast, families in the high-chaos worsening class (25%) experienced the most dysfunctional family environments, which further became worse after the COVID pandemic. This might relate to families’ economic status, as previous research has suggested that economic hardship is positively associated with HHChaos. Families with greater economic resources and stability might be better equipped to buffer disruptions within their household environments, whereas economic hardship may exacerbate HHChaos (53).

### Discrepancies in HHChaos trajectories between parents and youth

For the second research question, which examined differences in parents’ and youth's perceptions of HHChaos, we found notable evidence that parents and youth within the same family exhibited differing perceptions of HHChaos, with variations also observed between older and younger youth. For example, in dyadic families with one child, when parents experienced the low-chaos thriving trajectory, two-thirds of older youth (67%) reported being in the other two classes characterized by moderate or high chaos. Conversely, most younger youth (80%) perceived a similar low-chaos thriving trajectory as their parents. This disparity may stem from older youth assuming more adult-like roles and responsibilities during the COVID pandemic, potentially reducing the perceived stress for their parents and younger siblings (38). In addition, when parents experienced the high-chaos worsening trajectory, 43% of older youth and 67% of younger siblings reported a similar high-chaos trajectory. This pattern may reflect parents’ efforts to shield their children, as previous studies have shown that parenting behaviors can be a protective factor, buffering children's and youth's psychological responses to adversity events (54). However, younger children may remain more vulnerable to family-level stressors than older youth, given their less developed stress-regulation capacities and greater reliance on caregivers for coping (55). In triadic families (with two children), only 20% of parents and their two children experienced matching HHChaos trajectories throughout the COVID pandemic. These findings underscore the importance of creating opportunities for children, particularly older youth, to express their feelings and experiences, as they may perceive household conditions differently from their parents, even when families appear to be functioning well. Meanwhile, future interventions aimed at supporting families experiencing significant challenges should prioritize younger children, who may be more vulnerable to external stress. The more we prepare families to manage the demands of stressors (like a pandemic or other unanticipated disruptions), the more we can buffer young people from potential negative consequences.

### Relations to youth mental health and sleep problems

For the third question, which investigated the association between distinct HHChaos trajectories and youth health outcomes, our results align with prior literature. We found that youth from families experiencing high chaos showed higher levels of behavioral and emotional problems, sleep-disturbance, and lower levels of well-being compared to those from families in low-chaos thriving or moderate-chaos resilience classes (24–26). According to Bronfenbrenner's bioecological model of human development, HHChaos is harmful to youth because it interferes with interactions between children and their immediate family environment. Sustained interactions between parents and youth become difficult to maintain in chaotic environments (56). Specifically, a lack of routines may strain youth's self-regulatory ability. As routines are resources of feedback from the environment, it is difficult for youth to understand the consequences of the individual's and family's plans and expectations and further manage their own behaviors and emotions (57). Meanwhile, without structure and predictability, youth may undermine their sense of mastery and self-efficacy (56), leading to a high risk of emotional dysregulation, anxiety, stress, and difficulty in conducting proper behaviors. Conversely, living in a low-chaos family can provide children with a consistent and stable home environment, which benefits them in fostering a sense of competence and reducing behavioral and emotional risks (58). Moreover, the findings support previous research showing that greater HHChaos was associated with worse behavioral and physical health in youth. In a chaotic home situation, youth may feel helpless and incompetent, which may compromise their physical and behavioral health. As for sleep disturbance, youth in homes characterized by family chaos and disorganization may experience disturbance in sleep hygiene habits, such as inconsistent bed or waking times or difficulty accessing quiet sleep environments (26). Such disturbance, compounded by COVID pandemic-related stress, may increase cortisol production, a known predictor of insomnia (59). These irregular sleep patterns and difficulties can further contribute to sleep disturbance (60).

### Strengths and limitations

This study had several notable strengths. First, the overall sample size of 599 was quite large, enhancing statistic power of the main findings. Second, having multiple informant perspectives on household functioning provided a more comprehensive and ecologically valid assessment. By incorporating both parents and youth perspectives on constructs such as household chaos and youth behavioral problems, the study was able to capture both share and nonshared experiences within family system, which have been often overlooked in single-informant designs. Prior studies on COVID-19 pandemic have rarely included both parent and child reports, and even fewer studies have involved the reports of multiple children in the same family. The present study addresses these gaps by including reports from both parents and multiple youth within families. In addition, the use of multi-informant data allowed us to examine discrepancies between parent and youth reports, and between older-youth and younger-youth. These could provide valuable insights into differences in perception that may themselves be meaningful and could inform the design of more targeted intervention and family-level programming.

The findings of this study should be considered in light of its limitations. First, the youth subsample size was relatively small, limiting finding generalizability despite their value in capturing differences in perspectives between parents and youth in the same household. Larger-scale studies are needed to verify these family level patterns. Second, the study relied on retrospective accounts across the COVID pandemic due to the speed with which it unfolded. A prospective longitudinal study with multiple time points would be able to address this limitation and enable causal inferences about the relationship between HHChaos and youth development. Given how quickly the pandemic unfolded, this was not possible in this study, but a long-term longitudinal study would enable the effects of an unexpected systemic shock to be captured. Third, this study characterized the end of the COVID pandemic as 2,022, prior to the official declaration of the end of the public health emergency but after the reopening of many US schools; a longer-term follow-up may show additional recovery but would be more likely to be influenced by other factors. Finally, all the measures were based on parent- or youth self-reported, and youth outcomes were reported either parents or youth themselves. Although some of these outcomes could be measured physiologically (e.g., sleep with actographs) or through medical records (e.g., mental health diagnoses), these measures were not feasible for this study. Self-reported data are subject to social desirability bias, which could be partly explain discrepancies between parent and youth perceptions if one group is more biased than the other. Our interest in perceived lived experiences is best captured via self-report.

### Implications for public health and future pandemic preparedness and future directions

From a public health perspective, these findings highlight the importance of addressing household chaos as a key contextual risk factor for youth physical and mental health and well-being during large-scale disruptions such as COVID-19. Interventions should move beyond individual-level approaches and incorporate family-level strategies that promote stability, routines, and supportive caregiving environments, particularly for families experiencing high levels of chaos (56, 58). Given the observed discrepancies in perceptions within families and the differential vulnerability, where older youth appear more vulnerable in low-chaos contexts (as they demonstrated greater alignment with their parents’ classification within the low-chaos profile) and younger children in high-chaos contexts, public health programs should adopt developmentally tailored approaches, ensuring that different classes of youth experiences are recognized and addressed. Furthermore, future preparedness and planning should integrate family-centered strategies, such as access to economic resources, parental guidance, and community-based services to buffer the impact of stressors on the youth and the whole family's functioning (12, 38).

## Conclusion

Although we used a cross-sectional design, the current study still captured the distinct trajectories of HHChaos across the COVID-19 pandemic, narrowing the literature gap by providing the diversity of family classes. Notably, this research innovatively incorporates multi-method approaches, providing the youth perspectives and comparing the different perceptions between parents and youth within the family, even though the dyadic and triadic sample sizes were small. These findings can help future studies further explore the long-term impacts of HHChaos on youth health and well-being and provide targeted interventions for diverse family members.

## Data Availability

The datasets presented in this article are not readily available because to maintain confidentiality and privacy of the case study participant, data will remain protected. Additional information, however, can be made available from the corresponding author upon reasonable request. Requests to access the datasets should be directed to Jacinda K. Dariotis; dariotis@illinois.edu.
